# Users’ Experiences With Web-Based Health Care Information: Qualitative Study About Diabetes and Dementia Information Presented on a Governmental Website

**DOI:** 10.2196/11340

**Published:** 2019-07-08

**Authors:** Therese Agnes Wiegers, Michelle Hendriks, Uriëll Malanda, Dolf de Boer

**Affiliations:** 1 Nivel Utrecht Netherlands; 2 Reinier van Arkel 's Hertogenbosch Netherlands

**Keywords:** patients, qualitative research, choice behavior

## Abstract

**Background:**

Information on health and health care is abundant on the internet. To make informed choices, patients need reliable and easy-to-understand information about quality and availability of care providers and treatment options. However, the reliability of such Web-based information is difficult to assess.

**Objective:**

This study aimed to test Web-based information about diabetes and dementia and specifically a new presentation format of care routes to see if people are able to understand and use the information.

**Methods:**

Overall, 38 cognitive interviews were held; 20 people viewed the information about diabetes and 18 viewed the dementia information. Participants were asked what they would want to know about either diabetes or dementia, what choices they would want to make concerning their preferred care provider and treatment, and what information they would like to find to make these choices. They were then asked to view the relevant pages and comment on them. The interview was focused on general information about the condition, the care route, and the quality information for choosing a hospital. The interviews were transcribed verbatim and then systematically coded and ordered into themes.

**Results:**

The themes that were developed for both Web pages during the analysis were information needs, findability, usability, comprehension and readability, recognizability, care route, quality information, and usefulness. Information needs were found to be very diverse and dependent on the personal situation and condition of the participant. Several participants were unable to find specific items because they were not where they expected them to be. Most participants were positive about the layout, font, and color scheme of the test pages. However, options of clicking through to another website and indications where information can be expanded and collapsed could be made clearer. Participants generally found the information easy to understand but felt a need for a more detailed explanation of the medical terms. Recognition of the information played an important role: participants assessed whether the information they found matched their experiences. The term care route meant little to most of the participants, but the layout of the care route itself was found to be clear. Not many respondents spontaneously went to the quality information, and a number of participants had difficulty understanding it. Overall, the participants thought the information on the website was useful and helpful.

**Conclusions:**

The cognitive interviews gave numerous insights into how Web-based information is processed and understood. The care route offers a clear overview of the various stages as the condition progresses, but the name *care route* is not clear to everyone. We gained insight into differences between subgroups of people in terms of information needs, comprehension, and use of the information because the diversity within the group of participants was lower than expected.

## Introduction

### Background

Information on health and health care is abundant on the internet; it is present in all kinds of forms. Health information can be seen as information about diseases or health conditions; about signs and symptoms; about progression, severity, and duration; and about treatment possibilities. Health care information is more about care providers and care institutions, about accessibility and availability, about costs and quality, and about choosing a care provider or a treatment.

It is well understood that to be able to make an informed choice for a specific care provider or a specific treatment, patients need reliable and easy-to-understand information about the quality and availability of care providers and treatment options. However, the reliability of much of the information found on the internet is difficult to assess. In addition, the availability of information alone is no guaranty for its proper use. For instance, free provider choice and costs are relevant as well [1]. Governments or providers or insurers may be concerned about the reliability of the information available on the internet and may want to provide some kind of guidance to consumers looking for information. This may have 2 purposes: informing consumers about specific health issues by providing reliable and up-to-date medical information (providing health information) or informing consumers about quality issues to encourage them to make informed choices between care providers, care institutions, or treatment options (providing health care information or choice information). Websites may focus on one of these aims or may be designed for both uses. NHS Choices [2] is an example of a government website providing health information as well as choice information. A recent study by Lee et al has shown that there is a need among health information seekers for a centralized health portal or database containing relevant and quality health information [3].

In the previous 20-odd years, several studies have been conducted about the way information about health and health care could best be presented on the internet to make it accessible for anyone. According to Eysenbach [4], there are different levels of accessibility. The first level is physical accessibility: having access to the information, in this case, having a computer and having access to the internet. The second level is findability: finding the relevant website or finding information within a website. The third level is readability: is the language understandable for most people. The final level of accessibility is usability: the way the information is grouped and presented, how the user navigates through the information, and the amount of help the system gives.

Some studies focused on the information-seeking behavior of health consumers [5] or of health consumers and health professionals [6]. Pang et al discussed different search approaches in the process of health information seeking, depending on the level of knowledge about the health problem, level of curiosity, and perceived situational relevance to the health problem [7]. They state that people familiar with the health problem will show different seeking behavior than people not familiar with the condition. A review by Victoor et al [8] was focused on whether and how patients choose a provider. Other studies specifically focused on providing comparative health care information to consumers, with the intention to facilitate choice, such as American studies concerning decisions about which health plan to join [9,10,11]. As many of these studies have shown [12,13], the way choice information is presented is of utmost importance. Years earlier, Bettman and Kakkar [14] already examined the effect of information presentation format on consumers’ information acquisition strategies. They found that the strategies used to acquire information are strongly affected by the structure of the information presented. These results have implications for decisions on how to present information to consumers [14]. Their findings also apply to health care information.

Providing consumers with reliable medical information about specific health issues requires a different approach than providing choice information. Conflicting views and standards exist on how (and how much) medical information should be provided to consumers [4]. What is known is that people on average can process about 6 pieces of information at a time and are easily overwhelmed by information [15]. Research on how information is presented invariably shows that information on a website should be kept as simple as possible [12,13,16]. Information should be provided in layers, with a clear but concise overview on the main page, with links to additional information for those who are interested [16]. However, even with the information available, only a small number of people will use that to make an informed choice [1,8].

The Dutch government sees it as its responsibility to make understandable and reliable information about the quality and availability of health care in the Netherlands accessible for everyone. In 2004, the Dutch Ministry of Health commissioned the National Institute for Public Health and the Environment to develop, host, and manage a public national health and care portal on the internet, called KiesBeter, which translates as *Choose Better*. Its objectives were twofold: to improve the information position of consumers and to enable citizens to make informed decisions in health, health care, and health insurance. The portal seeks to provide reliable, independent, and coherent information on a range of health care–related topics [17]. Since 2014, the portal is hosted by the National Healthcare Institute of the Netherlands (Zorginstituut Nederland) [18], which is tasked with making information about the quality of care available to the public. The idea is that this portal will function as a starting point for health care consumers and will direct people through links to reliable and relevant information, provided by other sources, such as health care professionals, patient organizations, or health insurance companies. On the website, KiesBeter, people can find general information about health conditions, guidelines for good quality health care in general and for the appropriate care for any given condition, and comparative information to choose a health care provider or hospital [19].

Since the update in 2014, the website has experimented with providing information in the form of *care routes* for specific conditions. *Care routes* are not the same as *care pathways*. *Care pathways* are usually presented as a description of the necessary care for an average patient with a certain condition and are mostly aimed at health care providers [20]. The *care route*, as presented on KiesBeter, shows the trajectory of a typical patient through the health care system, from diagnosis to possible end stages as the illness progresses, with appropriate links to other websites with reliable information about the condition and treatment in that particular stage. Visually, a *care route* is presented as a kind of railway track, with stations representing access to specific information or available choices (see Multimedia Appendix 1). Thus, the *care route* combines health information and health care information. The aim of presenting *care routes* was to provide visitors of the KiesBeter website with insights into what good care is for a particular condition, what choices of treatment or of care providers they will encounter, and when they will encounter them and to encourage them to make their own choices based on high-quality information available on other websites.

Presenting *care routes* on the internet is a new phenomenon in Dutch health care and elsewhere. Little is known about the effectiveness and usability for health care consumers of information presented this way. In 2013, the Netherlands Institute for Health Services Research (NIVEL) conducted a study to find evidence in (international) literature that a *care route* is a useful format for encouraging people to choose between care providers [16]. The conclusion was that the proposed presentation of a *care route* on KiesBeter fits nicely with the requirements (as simple as possible, in layers, and with links to additional information) and may encourage patients to make informed choices about care providers [16].

### Objectives

Before actually implementing the *care route* for a number of conditions, an additional study was conducted to test the format among patients to see if they understand the information and are able to use it. In this paper, we present the results of that study carried out in 2015. For this study, information pages including *care routes* for dementia and diabetes were made available as test versions that were not yet available on the dementia and diabetes pages on the KiesBeter website. Cognitive interviews were held with a range of consumers, aiming to get a clear picture of users’ experiences with these pages on KiesBeter, with a focus on what information is sought (information needs) and how the information is understood (information comprehension).

The research answers the following questions:

1. What information do the majority of participants want to find and what information is only read by a few participants? (Information needs.)
2. How do participants read and process the information on the KiesBeter website, in particular, the *care routes* for dementia and diabetes? (Information comprehension.)

## Methods

### Participants

Participants were recruited through various channels with the aim of reaching different groups in society:

- NIVEL invited 246 members of the Dutch Health Care Consumer Panel to participate in a personal interview. The selection criteria were location (not too far from the place where the interviews were held), patient activation score, health literacy score, and education level. Members with a low patient activation score were oversampled. Familiarity with diabetes or dementia was not included in the selection criteria. This resulted in 11 interviews, 10 focusing on the diabetes page and 1 on the dementia page. Most of them were familiar with diabetes (6) or dementia (1), whereas this is unknown for the remaining 4.
- The Dutch Diabetes Association (DVN) invited people to participate via their website, their newsletter, and Twitter. This resulted in 10 interviews, all focusing on the diabetes page.
- Alzheimer Nederland sent 193 members of the Dutch Alzheimer Panel invitations by email for an interview. This panel consists of informal carers of people with dementia. This resulted in 17 interviews focusing on the dementia page.

We purposefully recruited participants from DVN and Alzheimer Nederland because they are familiar with the condition, so they would be motivated to look for information that would be important or relevant to them.

### Structure of the Diabetes and Dementia Web Pages

Each Web page opened with general information about the condition ordered in short statements or questions that could be expanded. One of those statements was *care route*. When expanding that statement, the *care route* became visible, divided in *stations*, which, in turn, could be expanded to reveal more information and link to a different website (see Multimedia Appendix 1). For diabetes, the main *stations* were diagnosis, first treatment phase, and chronic treatment phase. The route between the main stations showed additional stations. For instance, the stations after diagnosis were complaints, early detection, and elevated blood sugar value, each of which could be expanded for additional information. Following the statements was a text section called “Find a care provider,” which linked to another part of the website, where on the basis of distance (from a given position) and a choice of quality indicators (not obligatory), up to 3 hospitals could be selected. On the right-hand side of the page, in a different color, additional information was presented about the quality of care for the relevant condition and about the prevalence of the condition. On the diabetes page, the “Find a care provider” link was repeated here.

### Structure of the Cognitive Interview

In the interviews, before opening the Web page, the participants were first asked what they would want to know about either diabetes or dementia, what choices they would want to make concerning their preferred care provider and possible treatment, and what information they would like to find to make these choices. They were then asked to view the relevant KiesBeter pages and see if they could find the answers to those questions.

The interview consisted of 3 parts: part 1 was focused on the general information about diabetes or dementia, part 2 was focused on the *care route*, and part 3 was focused on the quality information for choosing a hospital. We used the thinking-aloud technique combined with verbal probing. The participants were asked to view the Web page as if they were at home and think out loud while doing so. They were invited to describe what they saw and to explain certain terms used and infographics presented on the website. Verbal probing was used to clarify how the information was being understood and interpreted and what might be missed. Each part of the interview and the interview as a whole was concluded by asking the participants if they had been able to find the information they were looking for, if any information was missing on the website, and if they had any suggestions for improvements. After each interview, the interviewers wrote down their impression of the conversation and any significant observations.

### Analyses

The interviews were transcribed verbatim and then systematically coded and ordered into themes by 2 researchers (MH and a junior researcher). The researchers conducted descriptive thematic analysis, consisting of an open coding and an axial coding phase: relevant themes were extracted, categorized, and classified. Then, relationships between categories were identified in the axial coding phase [21]. The results below are ordered according to these themes. Where relevant, a distinction is made by condition: diabetes or dementia.

## Results

### Participants

A total of 38 people participated in the cognitive interviews: 20 people, of whom 14 were diagnosed with diabetes or were familiar with the condition, viewed the page about diabetes and 18 people, all familiar with dementia, viewed the dementia page. The interviews were held at NIVEL or in people’s homes. Table 1 gives an overview of the participants. The average age was 65.7 years (ranging from 29 to 81; SD 10.7); 65% of them were older than 65 years. A little over half of them were women. The participants were highly educated compared with the general population; almost half of them had completed higher professional education or university education. One-third (32%) of the participants said their health was moderate or poor. Three-quarters of the participants had been using the internet for over 8 years, and 3 out of 4 of them used it daily. Less than half of the participants had visited the KiesBeter website before participating in the study. The majority of the participants considered themselves to be good with computers, felt confident in their use of the internet for collecting information, and said they used the internet to find information that was difficult to find elsewhere.

Our invitation policy had been aimed at different groups regarding the level of education, health literacy, patient activation, internet use, and health status. However, as Table 1 shows, the people responding turned out to be a selected group of, on average, highly educated, healthy, and confident internet users.

### General Impression

A finding from the interviewers’ observations was that it was difficult for the respondents to view all parts of the selected Web page within the time frame of 1 hour. This means that not all the elements of the Web page have always been addressed to their full depth. The website contained too much information to view and discuss in the allotted time. In addition, it was noted that the participants varied considerably in the extent in which they thought aloud when viewing the website. Some talked spontaneously about what they saw and thought of the website, whereas others needed more prompting. Their internet skills also varied, as did the way the participants browsed the website. Where some might systematically view the Web page from top to bottom and read all the available information, others would scroll quickly through the information and only read what they were interested in. Some people only looked at the information on KiesBeter, others also clicked through to other websites. Therefore, the general impression was that although the participants were a selected group regarding their education, health, and internet use, they were a very diverse group with regard to their behavior while viewing and commenting on the Web pages under study.

### Themes

The themes for both Web pages that were developed during the descriptive thematic analysis were: information needs, findability, usability, understanding and readability, recognizability, *care route*, quality information, and usefulness. Some of these themes (findability, usability, understanding and readability, and recognizability) closely resemble the earlier levels of accessibility of information described by Eysenbach [3].

#### Information Needs

The information needs were very diverse and dependent on the personal situation and condition of the participant. Some of the participants did not have a clear question at the time of the interview because they had been coping with the condition for years and already were familiar with most of the information. Several participants also mentioned that they already received all the information they wanted from their health care providers, such as their general practitioner, case manager, or specialist. Informal caregivers of people with dementia also regularly mentioned the Alzheimer Café and the Alzheimer Nederland website as sources of information:

Yes, actually I already know a lot about diabetes, so yeah. No, I keep up with new information, I pretty much already know the rest.

“What is dementia”...I’d skip that bit, because I think I already know.

**Table 1:** Characteristics of the cognitive interview participants for KiesBeter (N=38).

Demographic characteristic		Statistics, n (%)
**Gender**		
	Men	17 (45)
	Women	21 (55)
**Diabetes, dementia, or unknown**		
	Diabetes patient or familiar with diabetes	16 (42)
	Familiar with dementia	18 (47)
	Unknown	4 (11)
**Education**		
	Lower or prevocational secondary education	2 (5)
	General secondary education	5 (13)
	Senior secondary vocational education	7 (18)
	Senior general secondary education and preuniversity education	7 (18)
	Higher vocational education	9 (24)
	University education	8 (21)
**General health**		
	Excellent	1 (3)
	Very good	9 (24)
	Good	16 (42)
	Moderate	10 (26)
	Poor	2 (5)
**Years using the internet**		
	Less than 4	3 (8)
	4-6	2 (5)
	6-8	4 (11)
	More than 8	29 (76)
**Frequency of internet use**		
	Daily	28 (74)
	Several times a week	8 (21)
	Once a week	1 (3)
	Once a month	1 (3)
**Visited the KiesBeter website before**		
	No	21 (55)
	Yes	17 (45)
**I’m good with computers**		
	(Entirely) Disagree	4 (11)
	Neither agree nor disagree	10 (26)
	(Entirely) Agree	24 (63)
**I often surf the internet without really knowing what I’m looking for**		
	(Entirely) Disagree	24 (63)
	Neither agree nor disagree	8 (21)
	(Entirely) Agree	6 (16)
**I feel confident about using the internet to collect information**		
	(Entirely) Disagree	0 (0)
	Neither agree nor disagree	7 (19)
	(Entirely) Agree	29 (81)
**Using the internet lets me find information that would be difficult to find elsewhere**		
	(Entirely) Disagree	2 (5)
	Neither agree nor disagree	9 (24)
	(Entirely) Agree	26 (70)

##### Diabetes

Participants who viewed the Web page about diabetes said they wanted information about the cause of diabetes, the different types of diabetes, the early symptoms and the progress of the disease, what they can do themselves to stay in control of the condition, and the latest developments in treatment options. A number of participants said that it should be made clear and stated in several places that the information on the website is only about type 2 diabetes and not type 1 diabetes:

The symptoms of early diabetes are actually non-existent or very limited. I’d add that.

I’d like to know how you get diabetes and whether it is linked to genetics or it is something you might get because of your eating habits.

I would look at what the current treatment methods are and...do I need syringes right away or do I get pills? That kind of thing.

What I can do myself. How do I get a grip on diabetes. The healthy lifestyle.

Well, more about new developments in diabetes care or whether there is light in the darkness. Even though I’m sixty-nine.

##### Dementia

The informal carers of people with dementia were also interested in information about the causes of the condition, its various forms, and its progress. Other aspects they mentioned were how to deal with someone with dementia, where they can get support for caring for their relative, the problems they may encounter, and various types of living arrangements. The informal carers also wanted practical information about available medical devices (such as smart home devices), where certain types of assistance can be requested (such as contact information for the care institutions), and how much this costs:

I would actually like to know if it’s hereditary, because most of my dad’s side of the family have had dementia.

What types of dementia there are and...what consequences they have.

It tells you here what you can do yourself, but I think there should be a referral to an informal-care support centre near you.

What kinds of living arrangements are there? How do you get information about them?

#### Findability

The majority of participants said that they did not know about the KiesBeter website until they were invited to participate in this study. They also said that they probably would not have found it by searching the internet:

How do you find a website like this? I’d never type in “kies beter” [choose better] to find a website like this.

I still think that I’d advertise it a bit more. It could be advertised on TV or a flyer—if you want information, just have a look at this website.

With regard to being able to find information on the website, several participants were unable to find specific items because they were not where they expected them to be:

The diagnosis, yes...What symptoms do you have? The symptoms aren’t mentioned here; I would have expected that though.

This is what I expected to find in the ‘care route’. Yeah, it’s there, like I thought...but, it’s too far away and hidden.

Getting crisis assistance involved...But I wonder: shouldn’t that also be part of ‘living at home’, because that is when you might need this information.

#### Usability

Most participants were positive about the layout, font, and color scheme of the website. Some said they would like a bigger font that stayed big when they clicked through to other pages or subpages:

The layout’s fine. It’s not too complicated. It’s easy to read.

Yes, it’s nicely laid out. The colours are clear.

People appreciated the fact that the website used short texts and then referred elsewhere for more information. However, it could be made more obvious where there is the option of clicking through to another website and where information on this website can be expanded and collapsed. It is important that this is clear and uniform throughout the entire website:

I like this, these links too. I like it because you keep an overview and can go back.

I can get enough information now that you’ve shown me these click-through options, but I hadn’t noticed them myself.

I can see that those words are a lighter blue, but it’d be better if they were underlined or a different colour.

When clicking on a link to another website (eg, to DVN or Alzheimer Nederland), the participants noted that they did not arrive on pages with the specific information they were looking for. Often, the link was to a home page, and they again had to search for information on that website. There was also a need for more links to other websites or subpages.

At the right-hand side of the Web page, a link was available, leading to quality information under the heading “Find a care provider.” However, several participants did not see the “Find a care provider” block, mainly because people did not look at the right-hand side of the Web page. Some informal carers looked for the quality information in the *care route*, in the parts about the diagnosis, or in “When living at home becomes impossible.”

The layout of the *care route* itself was felt to be clear. However, it was not clear to everyone that the bullets could be expanded (ie, when clicking a bullet, that part of the route was unfolded to present more detailed information):

I like this bit. You can expand this, yeah. That’s nice and clear. And the text is legible, enough space between, and so forth.

Sometimes there’s a bit too much information on a single page.

That’s a bit cumbersome...That you can click the bullets. You could add ‘hide’ or ‘show’ next to them.

#### Understanding and Readability

The information was generally found to be easy to understand, but the participants felt a need for a more detailed explanation of the medical terms. This could be done by adding a reference (link) or an information button. The quality aspects in the quality information section could also be explained in more detail:

You can clearly read what you can expect to happen. That’s something you should be reading, at any rate.

When they use terms I’d like them to be explained.

These words are so complicated, all those medical terms.

Some participants felt the language was too distant and too technical:

The entire tone of the text…it turns me right off, to be honest. I don’t like it at all, I feel like I’m being treated a bit condescendingly.

It’s more professional carer language, rather than patient language.

#### Recognizability

For both type 2 diabetes and dementia, recognition of the information on the website played an important role in the interview. When looking at the website, the participants assessed whether the information they found matched their experiences. This theme was most prominent among those looking at the pages about dementia. A number of participants said that, in their view, the information was too positive and did not give a realistic representation of the actual situation:

You realise that something is wrong because you have to urinate a lot. And very thirsty... that’s all true. And other symptoms can arise. That’s right enough.

diabetes

You keep talking about the GP here, but nowadays the diabetes nurse or the GP’s medical assistant also do it.

diabetes

Lots of facts. But it also generates expectations that can’t always be realised in practice. (dementia)

You could also describe what the real situation is like. That you just say that this will sometimes not be possible.

dementia

#### Care Route

The term *care route* meant little to most of the participants, especially to those who viewed the diabetes pages. It was more likely to be associated with different types of care providers than with different stages of the condition. However, some of the participants who viewed the pages about dementia did associate the term with the various stages of dementia:

I expected a route to the care institutions. Via the symptoms of dementia and how you get to the institutions.

What I think of when I hear “care route”, the name says it all...Distinguishing between early dementia and the middle and end phases.

I know the route by bicycle. I think it’s the same kind of route...but walking through the hospital for the care you’re going to receive

diabetes

#### Quality Information

Not many respondents spontaneously went to the quality information at the right-hand side of the diabetes page or within the *care route* of both conditions, and even fewer of them were really interested in it. When asked how they would choose a hospital, it turned out that most respondents made a choice based on distance, personal experience, and the GP’s advice. Some participants did not realize that they could choose their own care provider, until we asked them to. They assumed the situation would dictate which care provider they would see:

Well, I think that I’d look at what’s closest to me and ask the GP who they’d recommend if I don’t know any of the doctors.

You don’t have a choice, you find yourself in a situation, depending on the treatment.

And then I can see on the website what they can offer? Oh yes. I think that’s pretty good.

Most participants viewed the results of just 1 hospital, the closest hospital or the one they knew. Only a few participants saw that they could also select and compare multiple hospitals at once. When looking at the quality information, some participants did not realize that they could scroll down, as a result, failing to see information further down the page. Participants would have liked a link to the hospital’s own website.

A number of participants had difficulty in understanding the quality information. This mainly concerned the numerical information (for instance, number of patients treated in the index year and percentage of patients who needed acute hospitalization), but some medical terms also needed more explaining. Some participants pointed out that it was important that the information was up to date. One participant also said that the *star rating* information (based on a client questionnaire score, the CQi) of the customer experience (for diabetes) was not very informative. They were more interested in reading the stories of previous patients in their own words:

It says here: The number of patients treated is 920, why is that important? Is the maximum 300 per physician or something like that? I don’t know, no clue what this is supposed to mean.

diabetes

I’d be more interested in this...sure, what were the experiences of other people, but also: where was it measured, how many people…? What is Miletus?

diabetes, referring to a quality programme run by Dutch insurers

##### Diabetes

A number of participants who viewed the Web page about diabetes felt that information was missing about the distance to the hospital and the waiting times. In addition, a variety of items were mentioned that people would like information about when choosing a hospital. For instance, about the number of examinations on a single day, whether it is possible to make an appointment with a diabetes nurse, the expertise of the specialists, and how much time a care provider has for a patient.

##### Dementia

Informal carers of people with dementia felt that the most important aspects when choosing a hospital were personal attention, the content of the diagnosis consultation, the expertise of the medical specialists, and waiting times. However, rather than comparing hospitals, participants were more interested in comparing nursing homes, although that information was not available on these pages. For them, relevant aspects, besides information about the various types of living arrangements, were personal attention and quality of care in a nursing home. However, several of the informal carers said that information on the internet has virtually no influence on their choice of a nursing home. They would visit various homes and make a choice based on their own impressions and perceptions of the premises and its surroundings.

#### Usefulness

The participants thought the information on KiesBeter was useful and helpful but not so much for them personally. They stated that the website is primarily suitable for informing and reassuring people who have just encountered the condition:

To let people know what type 2 diabetes is and how to deal with it.

It’s to give people reassurance. That there are enough options in the Netherlands for coping with it.

I thought this information was very basic, for people who have just encountered the problem.

The website made people think:

Sometimes losing weight is so effective that you don’t need medicine anymore. That was news to me. I’ve never heard that.

diabetes

I thought it was useful to know all these numbers, stuff that you have to check daily.

diabetes

Interesting. It gets you to think and see whether we want a careplan, depending on how you define it

dementia

The participants thought the information was too general to apply to their own situation though. With regard to diabetes, it was the lifestyle information in particular that was too general, and for dementia, it was the information about how to relate to their relative:

Enough exercise, move about for half an hour. Well, “move about” is a little vague. What does that mean?

diabetes

Look here, it says you have to make sure your blood glucose doesn’t get too high, but it doesn’t say how.

diabetes

It doesn’t say anywhere what to do if someone doesn’t want to cooperate.

dementia

What it says is nice, but you’ve got to be able to do it yourself. It’s more like suggestions, nothing very concrete.

dementia

## Discussion

### Overview

The aim of this study was to gain insight into how people process health and health care information on a website such as KiesBeter, especially the information concerning the *care route*, and to learn how a website like this could be improved. We had 38 participants think out loud when viewing the website, with verbal probing at specific moments. These participants were not *random* people, but most of them were familiar with the condition, being diabetes patient themselves or knowing someone with diabetes or being an informal carer for someone with dementia. We purposefully recruited participants familiar with these conditions and asked them before opening the Web page what they would want to know, so they would engage in a focused search. For this study, *care routes* were presented on test pages about diabetes and dementia but not available on the website. However, apart from the *care routes*, the pages were identical to the ones available on the website. Overall, 2 research questions were addressed in this study relating to information needs and information comprehension, including the *care route*. Cognitive interviews were held to better understand how people search for information and how they understand the information they find.

### Information Needs

Regarding health information, most participants were interested in the cause of the condition, the various forms, the way the condition progresses, and the consequences for daily life. However, the information on the website was too basic for many and was more appropriate for newly diagnosed patients. What the participants were looking for was more practical information that can be applied in day-to-day situations. Regarding health care information, no one expressed a need for quality, choice, or health care. The majority of respondents only looked at the available choice information after explicit prompting by the researcher. The finding that patients’ own impression and perception often impact their choice of health care more than information on the Web is supported by Victoor et al [8].

### Information Comprehension

Before comprehending information, it has to be accessible. The first level of accessibility described by Eysenbach [3], physical accessibility, is not relevant here because we provided the participants with access to a computer and to the website. The other levels, findability, readability, and usability are included under this heading of information comprehension. *Recognizability* is a new theme that emerged from the analysis, which may be rooted in the fact that most participants were familiar with the condition they were reviewing. In our view, this reflects the reality that information about certain conditions will primarily be sought by patients with that condition. In that respect, *recognizability* is an aspect of usability, because for that group of consumers, the information is usable.

The *care route* is a newly developed presentation format showing the trajectory of a patient from diagnosis through various stages as the illness progresses, thereby, providing health information about the condition as well as health care information about different care providers. The added value of presenting information in the form of a *care route* is its scope: all available themes are visible at the same time as *stations* in the route, with the option to expand each station. For a focused search, it is immediately clear where to look for more detail, while it reminds users of other aspects of the condition they might otherwise overlook. That way, it helps users in finding information that they were not looking for (serendipity) and may trigger their curiosity, as is described by Pang et al [7].

The *care route* was received well as a format, although the name *care route* was found to be somewhat confusing. However, the information provided in the *care route* did not fit in very well with the actual situation and the information needs of the participants, partly because they were already familiar with the general information and felt that concrete and practical information, which they could apply to their personal situation, was missing. The participants liked that only a summary of the various stages was given first, so that they could then choose which stage they wanted to know more about. However, it was not clear to everyone that more information about the different stages as the illness progresses was available (that the bullets or *stations* in the *care route* could be expanded).

We found that a small majority of the participants (55%) were not familiar with the website KiesBeter and thought they would not be able to find it by themselves. Some also had problems finding specific items on the website because they were not where they expected them to be (findability/usability). The participants thought the information on the test pages was generally easy to understand and clearly presented. They were positive about the layout and design (usability). However, there were some concerns about the language and about the visibility of click-through options: the (medical) terms used could be explained better and the click-through options to additional information could be shown more clearly and consistently (readability). The website, with its short texts and click-through options for more information, was found to be suited for all kinds of visitors, those reading from top to bottom as well as those scanning the page for interesting items. However, for the participants, who have been coping with the condition for several years, most of the information was already known. Therefore, these pages are foremost seen as a good place to start for people who are only recently diagnosed with the condition (usability).

The quality information or choice information was not seen at all by some and not seen as relevant by most. Participants were not really interested in information about the quality of care providers, presented as a comparison between health care institutions. They made their choice based on distance, personal experience, and their GP’s advice. This result corresponds with the findings by Victoor et al [8], who found that many patients make no active choice or choose a provider that is good enough based on only a few characteristics. In addition, we found that some of the information was poorly understood and the layout was not inviting enough for people to look at. The majority stated that they would sooner make a choice based on their own impressions than on information presented on the website.

### Limitations

The strength of our study lies in the richness of information we collected through the cognitive interviews. Most of our participants were able to engage in a focused search because they were already familiar with the conditions under study. A limitation was that the diversity within the group of participants was lower than expected. The people responding to our invitation turned out to be a selected group of, on average, highly educated, healthy, and confident internet users, as is often the case in-patient studies. Therefore, our results cannot be generalized to the general public. Future work should include a wider diversity of participants. Another limitation of our study, like many other studies on choice, for instance, the Zwijnenberg study [22], is that participants did not need to make a choice for a health care provider. The choice was hypothetical and therefore less relevant. It is unclear how people would choose in real situations. Nevertheless, we believe our results contribute to the knowledge on the design and the user experience of consumer health websites.

### Comparison With Other Studies

The themes resulting from the analysis closely resembled the levels described by Eysenbach [4]. This study confirms that information on a website should be kept as simple as possible [12,13,16]. The results about choice information are in line with the results Hibbart and Gutacker and Damman et al presented [11,12]. Zwijnenberg et al [22] stress the need for flexible, user-friendly websites or *information on demand*. With the presentation of the *care route* with all stages of the condition available in 1 overview and the option to expand each station for more information, the developers have aimed at just that. Furthermore, people told us they chose a hospital based on distance, personal experience, and their GP’s advice. This is also in line with the results by Victoor et al [8]. Zwijnenberg et al [22] explored patients’ preferences regarding the way comparative information is presented and the value of tailoring information to specific groups, but their group of participants, like ours, was not diverse enough to lead to a conclusion.

### Implications for Practice

The health information on the reviewed Web pages was found to be generally easy to understand and clearly presented, but the layout of the pages could be improved. For visitors familiar with the condition on the Web page, the information was too general. They would prefer to find more specific information that is applicable to their daily life.

One of the aims of presenting quality information is to encourage people to choose a care provider by comparing health care institutions, but we found that most people were not inclined to do so but rather would make a choice based on distance, personal experience, and the GP’s advice. Maybe focusing on choice is not the best possible approach when the ultimate goal is accessible and affordable health care. Maybe providing relevant and usable health information in itself will guide consumers’ choices enough to move toward that goal.

Our findings apply to the KiesBeter website but are relevant for other consumer websites as well. We conclude that it is important to involve future users in designing a health care information website, for instance, by conducting cognitive interviews as we did. This may help improve the quality and the usability of the website, preferably before it is implemented.

### Conclusions

The cognitive interviews gave numerous insights into how information on a website such as KiesBeter is processed and understood. The study indicates that for these respondents, who have been coping with the condition for several years, the added value of KiesBeter is small. Their impression is that it is more appropriate for people who have only recently been diagnosed with a condition and are looking for basic information about that condition. Providing choice information on a website does not seem to influence the way people make choices concerning a hospital. In addition, it seems not to encourage people to make an informed choice for a care provider. Finally, we observed that the name *care route* is not clear to everyone. On the other hand, the *care route* offers a clear overview of the various stages as the condition progresses.

## Appendix

Multimedia Appendix 1 Screenshot of KiesBeter, January 2014, Diabetes.

## Abbreviations

DVN: Dutch Diabetes Association
NIVEL: Netherlands Institute for Health Services Research
