# Supplementary material for: Users’ Experiences With Web-Based Health Care Information: Qualitative Study About Diabetes and Dementia Information Presented on a Governmental Website
Source: J Med Internet Res. 2019 Jul 8;21(7):e11340. doi: 10.2196/11340 (PMC6643759; doi:10.2196/11340)

Appendix A: Screenshot KiesBeter   January 2014, Diabetes

The page shows a number of statements or questions: What is Diabetes? Different kinds of Diabetes; How do you get Diabetes type 2? Progression of Diabetes type 2; Care route.

Each theme can be expanded with the blue button to find more information. After clicking that button on the last theme the ‘care route’ (‘zorgroute’) is presented as a blue line, with ‘stations’ showing details and links. The main stations are: Diagnosis, First treatment phase and Chronic treatment phase. The route between the main stations shows additional stations which can be expanded, for instance, after Diagnosis: complaints, early detection and elevated blood sugar value.

On the right hand side of the page general information is presented about good care in case of Diabetes type 2, followed by ‘Find a care provider’ with a choice for hospitals or home care organizations. Finally, the figure shows the number of people in the Netherlands (5 out of 100) with the diagnosis Diabetes type 2 (in 2014).

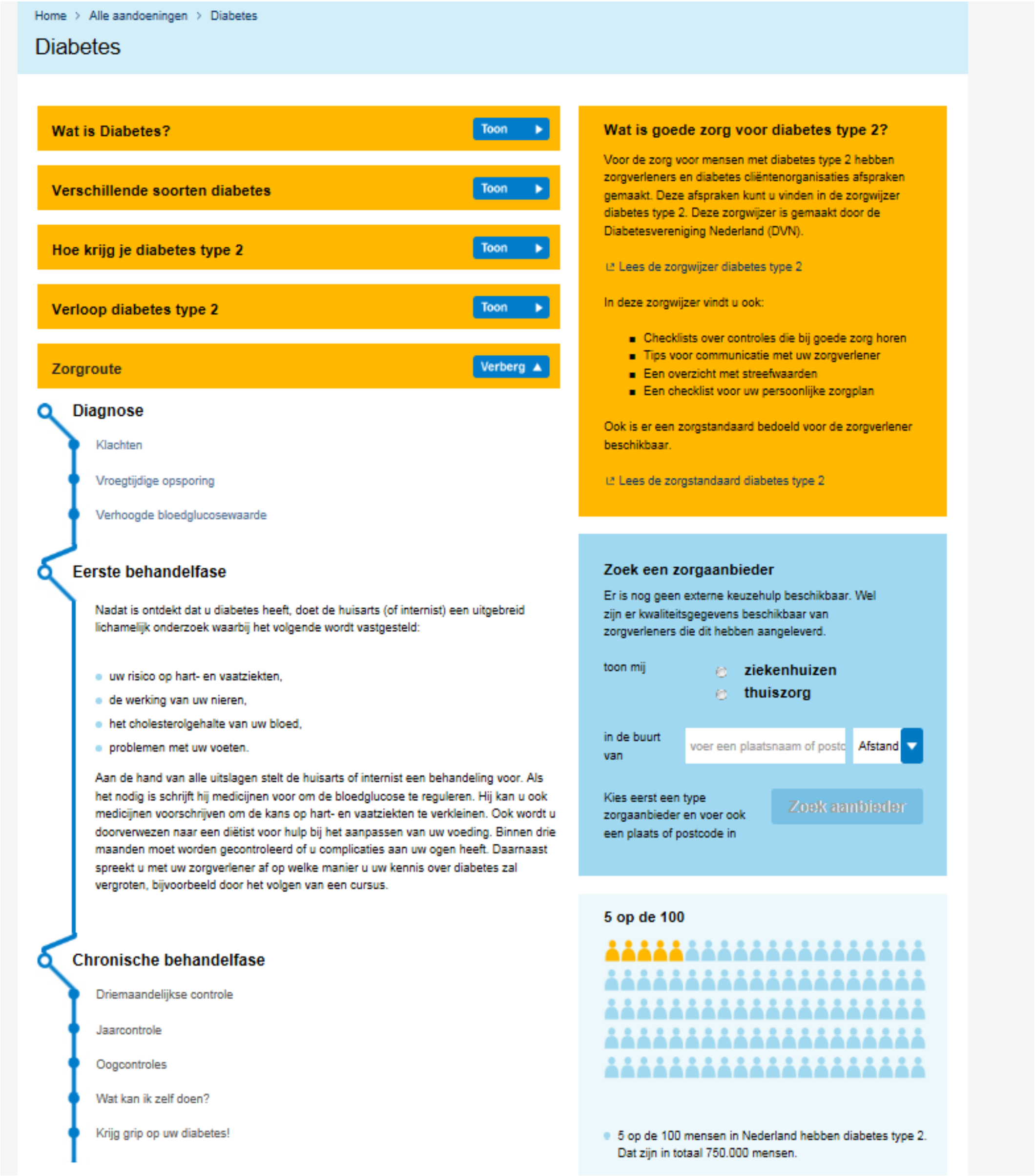

Supplement: Multimedia Appendix 1 [file jmir_v21i7e11340_app1.pdf]
